# Multiple species‐specific molecular markers using nanofluidic array as a tool to detect prey DNA from carnivore scats

**DOI:** 10.1002/ece3.7918

**Published:** 2021-08-01

**Authors:** Cecilia Di Bernardi, Camilla Wikenros, Eva Hedmark, Luigi Boitani, Paolo Ciucci, Håkan Sand, Mikael Åkesson

**Affiliations:** ^1^ Department of Biology and Biotechnologies “Charles Darwin” University of Rome La Sapienza Rome Italy; ^2^ Grimsö Wildlife Research Station Department of Ecology Swedish University of Agricultural Sciences Riddarhyttan Sweden

**Keywords:** *Canis lupus*, carnivore, cytochrome *b* gene, diet assessment, prey identification, species detection

## Abstract

Large carnivore feeding ecology plays a crucial role for management and conservation for predators and their prey. One of the keys to this kind of research is to identify the species composition in the predator diet, for example, prey determination from scat content. DNA‐based methods applied to detect prey in predators’ scats are viable alternatives to traditional macroscopic approaches, showing an increased reliability and higher prey detection rate. Here, we developed a molecular method for prey species identification in wolf (*Canis lupus*) scats using multiple species‐specific marker loci on the cytochrome *b* gene for 18 target species. The final panel consisted of 80 assays, with a minimum of four markers per target species, and that amplified specifically when using a high‐throughput Nanofluidic array technology (Fluidigm Inc.). As a practical example, we applied the method to identify target prey species DNA in 80 wolf scats collected in Sweden. Depending on the number of amplifying markers required to obtain a positive species call in a scat, the success in determining at least one prey species from the scats ranged from 44% to 92%. Although we highlight the need to evaluate the optimal number of markers for sensitive target species detection, the developed method is a fast and cost‐efficient tool for prey identification in wolf scats and it also has the potential to be further developed and applied to other areas and large carnivores as well.

## Graphical abstract

Large carnivore feeding ecology plays a crucial role for management and conservation for both the predators and their prey, and the identification of species composition in the predator diet is pivotal. We developed a molecular method for the identification of 18 target species in wolf (*Canis lupus*) scats using multiple species‐specific marker loci on the cytochrome b gene, which amplified specifically when using a nanofluidic array technology (Fluidigm Inc.). The developed method is a fast and cost‐efficient tool for prey detection in wolf scats and has the potential to be further developed and applied to other areas and other large carnivores as well.

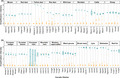

## 1 INTRODUCTION

Understanding of species’ feeding ecology is of critical importance when studying species interactions such as predator–prey dynamics (Symondson, 2002), and it can be a crucial tool to inform management and conservation (Newsome et al., 2016; Xiong et al., 2017). For large carnivores, collecting dietary information is difficult, since they are elusive and move over large areas (Kéry et al., 2011; Shehzad et al., 2012). In the field, GPS‐collars on predators have long been used to investigate predatory behavior and determine diet composition based on identification of kill sites through cluster checks (Peterson & Ciucci, 2003; Sand et al., 2005). One drawback of this approach is the potential bias against small prey species that require shorter handling times and leave few traces on kill sites (Bacon et al., 2011; Knopff et al., 2009; Webb et al., 2008). Scat analysis is a well established and frequently used methodology to characterize the diet of carnivores (Klare et al., 2011), with the advantage of being a noninvasive approach. Compared to GPS data, it is also more affordable when applied over large spatial and temporal scales. However, macroscopic scat analysis can present technical and interpretational challenges, such as various sources of bias in detecting and quantifying prey types and relative occurrence (Ciucci et al., 1996; Klare et al., 2011; Spaulding et al., 2000).

DNA‐based detection of prey from predators’ scats or guts has become a viable alternative for the analysis of food habits among invertebrate and vertebrate organisms (King et al., 2008; Pompanon et al., 2012; Traugott et al., 2021; Valentini et al., 2009). When compared with traditional morphological/macroscopic techniques, DNA analyses of scats have become more and more reliable due to a markedly higher prey detection rate (Casper et al., 2007; Mumma et al., 2016; Shores et al., 2015), a reduced observer bias (Shores et al., 2015), and a higher taxonomic resolution with more reliable separation of closely related taxa (Gosselin et al., 2017; Nørgaard et al., 2021; Shores et al., 2015). The predominant DNA region used for species discrimination in taxonomic and phylogenetic studies is the mitochondrial DNA (Simon et al., 2006), which, compared to the nuclear DNA, presents gene sequences with little intraspecific variability but provides adequate interspecific variation (Yang et al., 2014). Moreover, since the mitochondrial genome is normally represented in many more copies per cell than the nuclear genome, it has a greater chance of being amplified with PCR when samples contain few cells or degraded DNA (Yang et al., 2014). Within the mitochondrial genome, the cytochrome *b* (cyt *b*) gene is a suitable gene for species identification, being accurate in separating species and reconstructing phylogeny (Tobe et al., 2009, 2010).

When investigating generalist species or predators with unknown diets, universal primers followed by DNA sequencing have been frequently used in both vertebrates (De Barba et al., 2014; Jarman et al., 2013; Shutt et al., 2020; Šturm et al., 2021) and invertebrates (Pons, 2006; Symondson, 2002). Metabarcoding with next‐generation sequencing allows for high‐throughput identification of several species by simultaneously sequencing DNA from multiple species in environmental samples (eDNA; Francioli et al., 2021; Taberlet et al., 2012). In prey detection with generic primers, the amplifiable host DNA can however largely outnumber the presence of prey DNA (Krehenwinkel et al., 2017), and strategies to prevent host DNA amplification may be necessary, for example, by using predator‐specific blocking primers (Krehenwinkel et al., 2019; Shi et al., 2021; Vestheim & Jarman, 2008). When the diet is characterized by a limited number of prey species, and there is a priori knowledge of the animal's diet, multiplex PCR assays and DNA barcoding with species‐/group‐specific primers have been used, mostly in invertebrates (Harper et al., 2005; King et al., 2010, 2011; Staudacher et al., 2016) but also in mammals and birds (Casper et al., 2007; Deagle et al., 2007; Shores et al., 2015). Diagnostic PCR methods using species‐specific primers often involve relatively low cost per sample and are well suited for scats that contain multiple prey species, as detectability of a species in principal does not depend on the relative quantity of DNA from other species (Rubbmark et al., 2019).

The advances of nanotechnology and the multiplexing approach have improved the speed and efficiency compared to the more conventional PCR setups by reduced reaction volumes, number of pipetting steps, and a multiplexed preparation of DNA templates (Gorgannezhad et al., 2019; Wang et al., 2009). In particular, the use of Nanofluidic array technology (Fluidigm Inc.), which allows for multiplexing and high‐throughput analysis of small quantities of DNA, has proven to be useful for determining ungulate species from browsed twigs (Nichols & Spong, 2017) and blood samples (Blåhed et al., 2018), and for detecting pathogen species in ticks (Michelet et al., 2014). Moreover, nanofluidic array technology has also increased the efficiency of species and individual identification using single nucleotide polymorphisms (SNPs) of predator species from scat samples (Förster et al., 2018; Kraus et al., 2015; Von Thaden et al., 2017). Whereas this technology has increasingly been used as diagnostic tool for species detection, there is poor knowledge on its applicability to detect and identify prey DNA from predator scats using diagnostic molecular markers. The cost of using this technology in the year 2021 was ca 20 €/sample including DNA extraction. This aspect, together with the high sensitivity of detection when amplifying very short DNA fragment lengths (Broquet et al., 2007), potentially makes nanofluidic array technology a good contender to, for example, metabarcoding with NGS and conventional sequencing (Tercel et al., 2021) for detecting prey species from large sample sizes for ecological studies.

The aim of our study was to develop a molecular method using nanofluidic array technology with species‐specific molecular markers on the mitochondrial cyt *b* gene, for prey species identification in wolf (*Canis lupus*) scats for 14 potential prey species and four other carnivores in Scandinavia. Here, wild ungulates such as moose (*Alces alces*) and roe deer (*Capreolus capreolus*) represent the bulk of wolves’ diet (Sand et al., 2005, 2008). However, an expansion of the Scandinavian wolf population into habitats having multiple prey species, such as wild boar (*Sus scrofa*), red deer (*Cervus elaphus*), and fallow deer (*Dama dama*), would likely affect the predation ecology of wolves. As an example of the method applicability, we used our prey species detection procedure on a set of wolf scats collected within the genetic monitoring of the Scandinavian wolf population (Åkesson et al., 2016; Liberg et al., 2012).

## 2 MATERIAL AND METHODS

### 2.1 Development of molecular markers and target specificity test

We developed species‐specific molecular markers for 18 target species (moose, red deer, fallow deer, roe deer, wild boar, reindeer (*Rangifer rangifer*), sheep (*Ovis orientalis*), cattle (*Bos taurus*), European badger (*Meles meles*), Eurasian beaver (*Castor fiber*), European hare (*Lepus europeus*), mountain hare (*Lepus timidus*), Western capercaillie (*Tetrao urogallus*), black grouse (*Lyrurus tetrix*), brown bear (*Ursus arctos*), Eurasian lynx (*Lynx lynx*), wolverine (*Gulo gulo*), and red fox (*Vulpes vulpes*). The target species were selected among known prey species and also from allopatric medium‐sized and large carnivore species to wolves in northern Europe (Chapron et al., 2014; Gade‐Jørgensen & Stagegaard, 2000; Nowak et al., 2011; Sand et al., 2008). The species‐specific markers were developed using sequences of the cytochrome *b* (cyt *b*) gene in the mitochondrial DNA from the 18 target species, and wolf and dog (*Canis familiaris*; 1–25 sequences/species) found in GenBank on the NCBI website (http://www.ncbi.nlm.nih.gov/; Appendix S1). After aligning the sequences in Geneious Prime 2019.0.4 (Biomatters, Ltd.), we screened visually and identified species‐specific cyt *b* target DNA sites (loci; Appendix S2) that showed no conspecific variation and were highly diagnostic in relation to all target species, dogs and wolves.

We aimed at increasing marker species specificity in two ways. First, we had a strong preference for markers with fully diagnostic nucleotide at the 3′ end of at least one of primer‐pairs. At three occasions, two different wild ungulate species carried the same nucleotide at the 3′ end and in no cases was it the same nucleotide as those in the wolves and dogs. Second, when designing assays using Fluidigm's custom assay design criteria we added the instruction to increase target specificity by placing locus‐specific primers in regions that appeared conserved among conspecifics while differentiated in relation to the other target species, wolves and dogs. The aim was to use the Fluidigm EP1™ system to detect presence or absence of the target specific DNA. For this, we used assays (SNPtype™ assays, Fluidigm Corp.) with one reverse primer and two forward primers with identical annealing sequences but with different SNPtype™ (Fluidigm Corp.) tail sequences for HEX and FAM fluorescence (SNPtype™‐HEX and SNPtype™‐FAM). This enabled us to use either the FAM or HEX signal to quantify the amplification intensity (see below). We did not include dogs among the target species as we did not succeed in developing markers that specifically amplified and separated dogs and wolves. Moreover, there was already a potential risk of a negative bias against wolf scats containing DNA from dogs, since these scats are difficult to link to individual wolves, due to the overlap in allelic composition among the two species.

We developed 207 assays (Appendix S3) with a minimum of four assays and different loci for each target species. Multiple assays for the same locus and species were occasionally developed when we found nonsufficient separation in amplification intensity between specific and nonspecific species, but we finally kept one assay per locus. We aimed to develop assays for at least four loci per target species in order to (a) increase the chance of detecting the target species in the event of some markers not amplifying due to low DNA quantity, (b) account for the possibility that we missed intraspecific variation that prohibits amplification for some marker, and (c) increase the target specificity in the case of markers not being fully diagnostic in relation to other species. For most species, we tested more than four loci and we continued to use those that showed the best separation between specific and nonspecific reference tissue samples.

**FIGURE 1:**
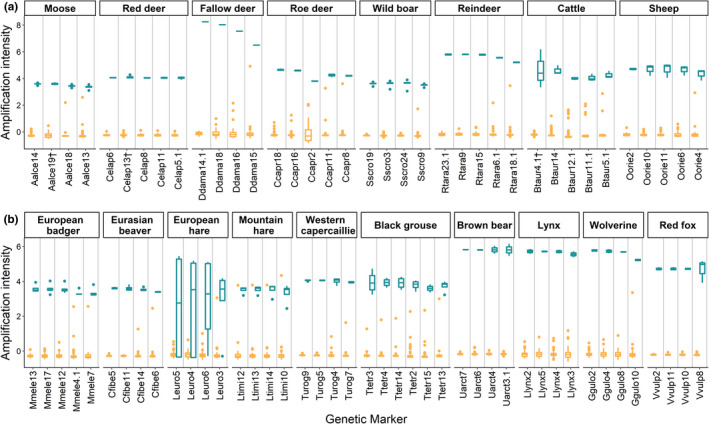
Amplification intensity for the specific (turquoise) and nonspecific (yellow) reference tissue samples analyzed on 80 different markers for the identification of (a) wild and domestic ungulates (moose, red deer, fallow deer, roe deer, wild boar, reindeer, cattle, sheep) and (b) smaller prey species (European badger, Eurasian beaver, European hare, mountain hare, Western capercaillie, black grouse) and large‐sized and medium‐sized carnivores (brown bear, Eurasian lynx, wolverine, red fox) A negative control (water) was used for each marker. The markers are arranged within each species based on the frequency of overlap and additionally on the distance between the minimum specific sample and the maximum nonspecific sample (from left to right, increasing frequency of overlap and decreasing distance). The amplification intensities were standardized for visual purposes. ^†^For 3 markers, we illustrate the amplification intensity from a rerun

The molecular markers were tested for target specificity with 2–5 tissue samples for each target species (≥3 samples for the wild and domestic ungulates), using specimens provided by the Swedish Museum of Natural History. The tissue samples were geographically distributed throughout Sweden in the attempt to cover any spatial intraspecific variability in sequences of the target species. Additionally, samples from wolves (*n* = 3), bank voles (*Myodes glareolus*; *n* = 5), and a negative control (water) were also included in the run. All the markers were tested against all the tissue samples. For each marker, a two‐sample *t*‐test was conducted between the amplification intensity of specific and nonspecific samples. Because of multiple testing, we adjusted the p‐values using the BY approach (Benjamini & Yekutieli, 2001). Additionally, the frequency of overlap was measured as the proportion of nonspecific samples overlapping in amplification intensity with the minimum amplification intensity of the specific reference tissue samples. Since the four fallow deer markers were tested with a one‐sample *t*‐test (only one specific sample was finally available for statistical analysis), we additionally ran a two‐sample *t*‐test and estimated the frequency of overlap from a rerun. Out of the 207 molecular markers developed and tested, we selected a final panel of 80 markers with the largest separation between specific and nonspecific reference tissue samples, maintaining a minimum of four markers for each target species (Figure 1, Appendix S3).

### 2.2 Molecular analysis and PCR optimization

DNA was extracted from the tissue samples using standard phenol/chloroform‐isoamylalcohol extraction, and DNA was quantified using NanoDrop™ 2000 Spectrophotometer. The prepared DNA (10 ng/μl) was amplified with PCR and visualized with fluorescence detection using a Fluidigm^®^ 96.96 Dynamic Integrated Fluidic Circuit (IFC) Array, according to the manufacturer's instructions (http://www.fluidigm.com). To avoid cross‐contamination, the PCR setup was done in a hood prepared with ultraviolet (UV) light exposure. Each Fluidigm plate enabled the PCR amplification of 96 assays on 96 samples simultaneously, and the standard procedure recommended by Fluidigm was modified by excluding the specific target amplification and increasing the starting temperature of the touch‐down cycle of 1℃ (65–60℃ with 1℃ decrease between cycles). Both modifications reduced the amplification intensity of nonspecific samples and therefore increased the specificity of our molecular markers.

Data on fluorescence intensity were obtained from the Fluidigm EP1™. The reported fluorescence signal, relative to the passive reference ROX™ dye, reflects the DNA amplification intensity (Kubista et al., 2006; Whitcombe et al., 1999). Since we used two fluorescence dyes on the same target locus, we got two measures of amplification intensity, *I_F_* and *I_H_*, respectively, representing the amplification intensity of SNPtype‐FAM and SNPtype‐HEX amplicons. To account for the overlap of amplification intensity between nonspecific and specific samples, which was occasionally observed in one of the two dyes of a marker, we systematically extrapolated the amplification intensity of the reference samples based on the frequency of overlap of both dyes. Frequency of overlap was measured for each dye as the proportion of nonspecific samples overlapping in amplification intensity with the minimum intensity of the specific reference tissue samples. If both dyes showed no overlap, one of the two was randomly picked; if only one dye had a null frequency of overlap, it was picked against the dye with frequency of overlap >0; if both dyes had frequency of overlap >0, the dye with lower frequency of overlap was picked. Occasionally, the ROX‐signal for some samples was near absent, possibly due to the occurrence of dirt particles in the samples that hindered the solution to flow in the IFC. As this appeared to affect the relative intensity of the calls, we omitted samples identified as outliers with regard to ROX intensity.

### 2.3 Wolf scat samples and target species determination

To exemplify the applicability of our method, we examined the occurrence of the target species in 80 wolf scat samples collected in Sweden between 2009 and 2018 (Appendix S6) during the yearly monitoring (October–March) of the Scandinavian wolf population (Åkesson et al., 2016; Liberg et al., 2012). The DNA was extracted within each monitoring period, using QIAamp DNA Stool Kit (Qiagen) or ISOLATE Faecal DNA Kit (Bioline). The presence of wolf‐specific DNA and the identity of the wolf were determined in accordance with the methods described in Åkesson et al. (2016).

We used thresholds for getting a binary detection for a prey species in each scat, where the intensity of 0.2 (value indicating low amplification intensity) and the intensities of nonspecific reference tissues from the run were used as baseline in each marker. Any sample showing intensities below the baseline was regarded as not amplifying. The sensitivity of using a minimum of 1, 2, 3, or 4 markers with a positive call (out of the total of used markers) for detecting the target species DNA was tested and compared (Appendix S7). For each such scenario, all the possible combinations of markers were checked, and the target species DNA was deemed as present in a sample when at least one combination showed amplification intensities above all markers’ baseline levels. This was done separately for the 18 target species. All statistical analyses were conducted in R version 3.6.0 (R Core Team, 2018).

## 3 RESULTS

### 3.1 Molecular markers

Amplification intensity of reference tissue samples for the target species varied from 0.02 to 1.31, with an average intensity of 0.93 (range 0.02–1.31) for specific samples and 0.08 (range 0.00–1.13) for nonspecific samples (Figure 1). The 80 selected markers all showed amplification for the specific samples, and the majority (*n* = 77) showed significantly higher intensity of specific than nonspecific samples (*p* ≤ 0.05, Appendix S4, Figure 1). After the correction for multiple testing, 70 out of the 80 markers had a significant separation (Appendix S4) and we hereafter refer to the adjusted p‐values. One moose marker (Aalce19) showed an overlap between specific and nonspecific samples with a higher intensity of the nonspecific samples compared to the specific samples (*t* = 3.83, *p* = 0.027, frequency of overlap = 1, Appendix S4). The 80 markers had an average frequency of overlap of 0.05 ± 0.02 (mean ± *SE*), with the majority (*n* = 71) having no overlap (Appendix S4). A sample from a morphologically determined European hare consistently amplified with markers for mountain hare, indicating that this individual had hybrid origin. After a rerun of the 11 markers with nonsignificant (*n* = 10) or negative difference between specific and nonspecific sample intensities (*n* = 1), we found that two out of 11 (Aalce19, Llynx2) showed significant separation with no overlap (Appendix S5). The other nine markers showed a nonsignificant separation in the rerun as well, with frequency of overlap >0.22 for the European hare markers and no overlap for the remaining markers (Appendix S5). For fallow deer, both the one‐sample *t*‐test and the two‐sample *t*‐test from a rerun resulted in significant separation and the frequency of overlap was zero for all four markers in both runs (Appendices S4 and S5).

For all target species, the final panel included at least four markers available for species identification, while five markers were available for red deer, roe deer, reindeer, sheep, cattle, and European badger, and six markers for black grouse (Figure 1, Appendix S3). The negative control never amplified with any of the 80 selected markers.

### 3.2 Application to wolf scat samples

**FIGURE 2:**
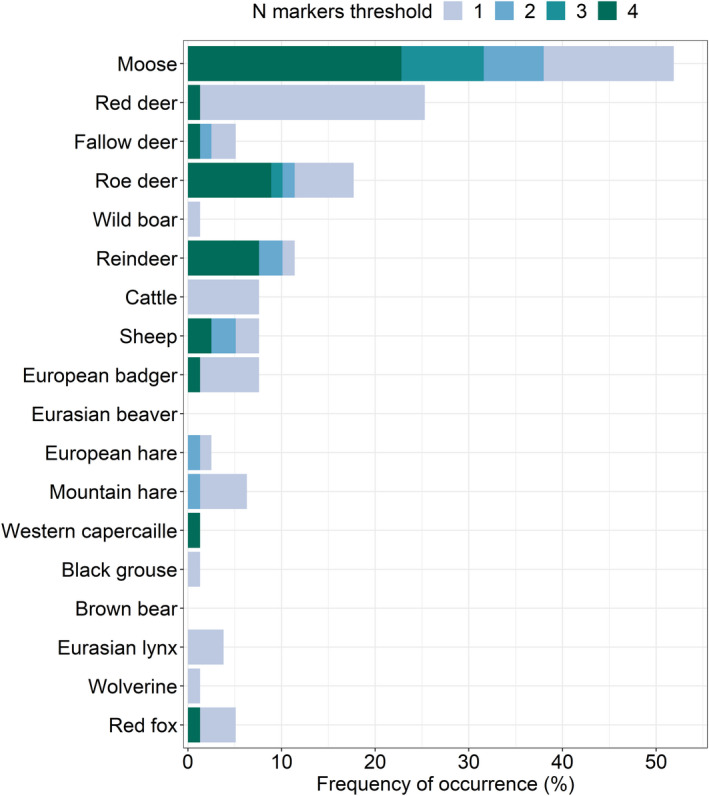
Prey diversity observed in the diet of wolves from scats (*n* = 79) collected in Sweden (2009–2018), depending on the threshold minimum number of amplifying markers required to detect the target species in a scat sample. The frequency of occurrence was measured as the percentage of scat samples with the detected target species out of the total number of samples analyzed. For the two hare species, the result illustrates the maternal lineages of the two species, while potential hybrid status of the detected hares was not known

Setting the thresholds to reach full specificity for each target species, we detected the presence of DNA from at least one target species in 73 (92%), 53 (67%), 43 (54%), and 35 scats (44%) when minimum one, two, three, and four amplifying markers were set as threshold, respectively (Figure 2). In each scenario, the remaining samples did not meet the criteria for species detection. Out of the 80 wolf scat samples analyzed, one was invalidated due to outlier ROX intensity. The average number of detected species per scat sample was, respectively, 1.7 (range 1–7), 1.1 (range 1–4), 1.1 (range 1–2), and 1.1 (range 1–2) when one, two, three, and four amplifying markers were set as threshold. In total, 16 different target species were identified, comprising wild ungulates (moose, red deer, fallow deer, roe deer, wild boar), domestic and semi‐domestic animals (reindeer, cattle, sheep), small prey species (European badger, European hare, mountain hare, Western capercaillie, black grouse), and other carnivores (Eurasian lynx, wolverine, red fox).

## 4 DISCUSSION

We developed a molecular method to detect prey species DNA in wolf scats by using multiple diagnostic molecular markers that amplified specifically when tested with high‐quality DNA, that is, tissue samples, from 18 target species. After setting thresholds that maximized specificity for a binary species detection, the application of the method to a sample of genetically verified wolf scats collected in the field was tested and resulted in the amplification of 16 species. While this study was not meant to make a comparative assessment between the nanofluidic array approach and traditional scat analysis techniques (i.e., hand separation), our aim was to develop a practical and efficient technique to identify prey species from predators’ scats and assess its performance.

The final panel contained 80 molecular markers with a minimum of four markers on different target loci of cyt *b* for each target species. Although the focal species we considered are wild ungulates, which make up the bulk of the diet in the Scandinavian wolf population (Sand et al., 2005, 2008), our panel also offers the possibility to detect smaller prey species that are less likely to be found by GPS technology due to their smaller amount of biomass (Sand et al., 2005). Markers for detecting other medium‐sized (red fox) and large (lynx, wolverine, brown bear) carnivores occurring in the study area were also included in the panel, and hold the potential for providing information on the interaction between wolves and other carnivores. However, is worth recommending that the presence of DNA from other carnivores does not necessarily indicate intraguild predation, as these species may be prone to contaminate scats with their DNA through territorial marking (Wikenros et al., 2017).

Among the 80 molecular markers we used, significant separation and low frequency of overlap generally indicated a good marker performance in discerning the target species. The hare markers successfully separated hares from the other target species, but the two different hare species were not always distinctly separated. Indeed, the consistent amplification of a morphologically determined European hare sample with mountain hare markers is likely due to hybridization, as the European hare and mountain hare hybridize in the wild (Jansson et al., 2007). We therefore caution against the distinction between the two hare species with only mitochondrial markers, but encourage to maintain the two developed marker sets separated in order to keep the distinction of maternal lineages. Beside the hare markers, for the few other cases of nonspecific samples with amplification intensity similar or higher than specific samples, a possible explanation could be that the nontarget species carried intraspecific variation that overlapped with the target species but was missing in the reference sequences used in this study (Appendix S1). Here, we took into account the nonspecific amplification by setting threshold intensities resulting in full specificity in relation to the range of tested reference tissue samples. This was done separately for the four scenarios, using a minimum of 1, 2, 3, or 4 markers with a positive call (out of the total of used markers) to determine the presence of target species DNA. When applying our molecular method to wolf scats, we obtained different DNA detection rates depending on the minimum number of markers required. The percentage of scat samples with presence of at least one target species was 92% when using a threshold of one marker, while it was 67% when using a threshold of two markers, therefore confirming the amplification on at least two independent loci. If we prioritize the sensitivity of our detection procedure and set a threshold of only one marker to detect a species, we minimize the occurrence of false negatives (type II error). However, despite that we developed markers as diagnostic as possible and set thresholds with full specificity in relation to the reference samples analyzed, the intraspecific locus variation in the wild may not have been fully represented among the animals in this study. As this can potentially lead to the risk of false positives (type I error), caution should therefore be taken with regard to using too few markers for a diagnostic species determination. In line with the principles of replication and multiple tubes approach (Ficetola et al., 2015; Taberlet et al., 1996), requiring more than one amplifying marker out of the used set of species‐specific markers may thus be a way to ensure the quality of target species determination.

Our diagnostic method with species‐specific markers adds to the more frequent studies using DNA from carnivore scats to identify prey (Hacker et al., 2021; Quéméré et al., 2021; Roffler et al., 2021; Shi et al., 2021; Smith et al., 2018; Xiong et al., 2017). These studies primarily used DNA metabarcoding, which produces a vast amount of valuable information but sometimes also needs consideration of potential bias sources in key steps in the data handling process, lack of reference databases of barcodes for many prey species, but also intensive laboratory procedures and considerable bioinformatics training (Hacker et al., 2021; Tercel et al., 2021; Zinger et al., 2019). The relatively simple molecular method developed here, applying the nanofluidic array technology to detect prey DNA, represents a promising and valid alternative to other methods. However, although the use of multiple markers has previously been shown to increase the species detection success (Zhang et al., 2018), further validation of our method by using scats with known content would provide further insights into the method sensitivity and which thresholds to use. The latter is indeed a critical step faced in other molecular approaches as well, including the setting of thresholds to discard sequences with next‐generation sequencing (Darling & Mahon, 2011; Taberlet et al., 2012).

As observed from our sample of wolf scats collected in Sweden, other studies using either traditional scat analysis and DNA approaches have shown that the majority of wolf scats contain on average one prey per scat sample (Ciucci et al., 2018; Shores et al., 2015). In addition, the occurrence of prey species detected in our study is in line with previous and ongoing research on the diet of the Scandinavian wolf population conducted using GPS technology. Specifically, moose and roe deer compose the bulk of the wolf diet in Scandinavia, but also predation on domestic animals (i.e., sheep and cattle) is evidenced (Karlsson & Johansson, 2010; Sand et al., 2005, 2008, 2016; Zimmermann et al., 2015). The position of the scats with detected large ungulate wild prey species, that is, moose, roe deer, red deer, and wild boar, fitted well within the species’ distribution range (Linnell et al., 2020). The detection of reindeer in wolf scats only occurred among scats collected within the reindeer husbandry area, in the northern part of Sweden, where wolf attacks on semi‐domestic reindeer are documented (Sand et al., 2019). Detections of fallow deer, red deer, and wild boar were found only in scats from the southern part of Sweden. This is where these species are known to occur, but there currently is little knowledge about their importance as prey for wolves. With the recent expansion of the wolf population into the southern parts of Sweden (Svensson et al., 2021), our method will therefore be a useful tool in investigating the potential changes in prey use of wolves and its effect on ungulate populations. Consumption of smaller prey has previously been documented by GPS technology as constituting a small percentage of wolves’ diet in Scandinavia (Sand et al., 2008; Zimmermann et al., 2015). However, GPS technology is likely underestimating the contribution of small prey, and DNA identification can contribute to better estimates of the frequency of small prey consumption by wolves. Moreover, the implementation of the molecular method to a broader sample of scats will increase our knowledge on wolf diet in areas that are difficult, or not prioritized, to cover with GPS‐collared wolves, for example, southern Sweden. The molecular method will therefore serve as a valuable complement to the current GPS technology used to investigate wolf predation.

Applications of our molecular method to the management of wolves’ main prey species include providing information about wolf prey consumption over large spatial and temporal scales. Knowledge of area specific wolf prey consumption, especially with multiple ungulate prey species, is important information for management when deciding on hunting quotas of ungulates. Additionally, this method can provide information on the use of domestic animals and thus help to set levels of compensation in areas with free‐ranging domestic animals (e.g., sheep in Norway, semi‐domestic reindeer in the reindeer husbandry area in Sweden).

We conclude that the method we developed, suitable for high‐throughput analysis of scat samples on up to 96 markers and 96 samples simultaneously, represents a promising noninvasive, fast, and cost‐efficient DNA‐based tool for ecological studies on wolves. As this method can be easily adapted to new situations and customized to fit regional demands with new prey species, it has the potential to be further developed and applied to other areas and other large carnivores as well.

## CONFLICT OF INTEREST

None declared.

## AUTHOR CONTRIBUTION

**Cecilia Di Bernardi:** Conceptualization (lead); Data curation (lead); Formal analysis (lead); Investigation (lead); Methodology (lead); Writing‐original draft (lead); Writing‐review & editing (lead). **Camilla Wikenros:** Conceptualization (supporting); Funding acquisition (equal); Methodology (supporting); Project administration (lead); Supervision (equal); Writing‐original draft (supporting); Writing‐review & editing (equal). **Eva Hedmark:** Conceptualization (supporting); Methodology (supporting); Supervision (supporting); Writing‐original draft (supporting). **Luigi Boitani:** Conceptualization (supporting); Methodology (supporting); Supervision (equal); Writing‐original draft (supporting); Writing‐review & editing (supporting). **Paolo Ciucci:** Conceptualization (supporting); Funding acquisition (equal); Methodology (supporting); Supervision (equal); Writing‐original draft (supporting); Writing‐review & editing (equal). **Håkan Sand:** Conceptualization (supporting); Funding acquisition (equal); Methodology (supporting); Supervision (equal); Writing‐original draft (supporting); Writing‐review & editing (supporting). **Mikael Åkesson:** Conceptualization (lead); Data curation (supporting); Formal analysis (supporting); Funding acquisition (equal); Methodology (supporting); Supervision (equal); Writing‐original draft (supporting); Writing‐review & editing (equal).

## Data Availability

The data that support the findings of this study are available in the Supplemental Information of this article, available from the Zenodo Digital Repository DOI https://doi.org/10.5281/zenodo.5066742. Specifically, the NCBI accession numbers of DNA reference sequences, and their literature citations where applicable, are available in Appendix S1, while the DNA sequence information of the developed molecular markers is available in Appendices S2 and S3.
